# Evolution of Complex Maillard Chemical Reactions, Resolved in Time

**DOI:** 10.1038/s41598-017-03691-z

**Published:** 2017-06-12

**Authors:** Daniel Hemmler, Chloé Roullier-Gall, James W. Marshall, Michael Rychlik, Andrew J. Taylor, Philippe Schmitt-Kopplin

**Affiliations:** 1 Comprehensive Foodomics Platform, Analytical Food Chemistry, Technical University Munich, Alte Akademie 10, 85354 Freising, Germany; 2 Research Unit Analytical BioGeoChemistry (BGC), Helmholtz Zentrum München, Ingolstädter Landstrasse 1, 85764 Neuherberg, Germany; 3 The Waltham Centre for Pet Nutrition, Mars Petcare UK, Waltham-on-the-Wolds, Leicestershire, LE14 4RT United Kingdom

## Abstract

In this study, we monitored the thermal formation of early ribose-glycine Maillard reaction products over time by ion cyclotron resonance mass spectrometry. Here, we considered sugar decomposition (caramelization) apart from compounds that could only be produced in the presence of the amino acid. More than 300 intermediates as a result of the two initial reactants were found after ten hours (100 °C) to participate in the interplay of the Maillard reaction cascade. Despite the large numerical variety the majority of intermediates follow simple and repetitive reaction patterns. Dehydration, carbonyl cleavage, and redox reactions turned out to have a large impact on the diversity the Maillard reaction causes. Although the Amadori breakdown is considered as the main Maillard reaction pathway, other reactive intermediates, often of higher molecular weight than the Amadori rearrangement product, contribute to a large extent to the multitude of intermediates we observed.

## Introduction

For more than 100 years^1^, understanding the Maillard reaction (MR) has been of great interest in food science. The MR refers to a non-enzymatic reaction between reducing carbohydrates and amino compounds. It can be understood as a complex network of chemical reaction series, rather than a single reaction. Hundreds or even thousands of distinct Maillard reaction products (MRPs) are generated, in particular through thermal processing. Many of the molecules contribute significantly to the aroma, taste and color of food^2^. In 1953, Hodge divided the MR into three essential steps^3^: (i) In the initial phase the carbonyl moiety of a sugar condenses with an amino compound; (ii) the subsequent rearrangement and breakdown of the Amadori compound (intermediate phase) leads to a reaction cascade involving dehydration, deamination, Strecker degradation, and many other fragmentation steps^4^; and (iii) high-molecular weight and colored compounds are produced from the low molecular weight intermediates^3^. In order to control the MR to produce desired molecules (*e.g*. flavors, antioxidants) and to avoid loss in nutritional value of food, it is essential to understand this entire chemical “collective”.

Traditionally, MRPs are analyzed by targeted methods. However, in targeted approaches only a small set of known (or predicted) compounds are studied at the same time. The bulk of the many unknown compounds remain ignored. By comparison, non-targeted analytical methods aim to investigate the entire complexity of a sample and provide the opportunity to solve yet unanswered issues^5^. Ultrahigh-resolution ion cyclotron resonance mass spectrometry (FT-ICR-MS) was recently used in several studies to fingerprint, classify and describe the chemical composition of several foods^6^ and beverages^7–9^. Jeandet *et al*. identified 5-hydroxymethylfurfural (HMF) and other caramelization markers by a combination of non-targeted FT-ICR-MS and NMR in 170-year-old champagne found in a shipwreck in the Baltic Sea^10^. Golon *et al*. reported a considerably higher number of resolved analytes in heated sucrose-amino acid mixtures analyzed by FT-ICR-MS compared to TOF-MS^11^. Despite the growing number of non-targeted approaches, the majority of MRPs, produced even when only one sugar is heated together with a single amino acid, still remain unknown. Despite decades of structural studies on the MR, a comprehensive picture of the composition of the reaction system and coherences between the intermediates has not been resolved. The aim of this study was to use non-targeted analysis and data visualization to deliver more insights into the overall chemical changes occurring in a ribose-glycine reaction.

## Results and Discussion

**Figure 1:**
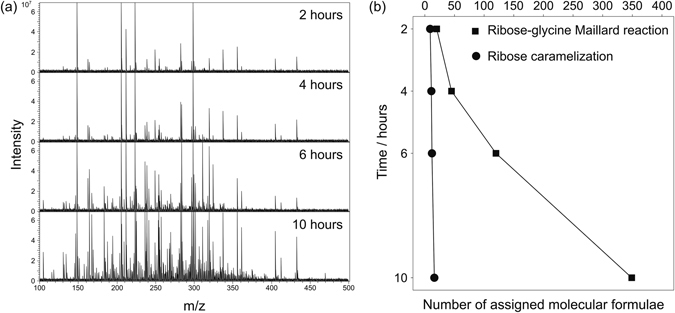
Progression of reaction products in a ribose-glycine Maillard model system after thermal treatment at 100 °C for two, four, six, and ten hours. (**a**) Raw FT-ICR mass spectra with fixed peak intensity scale. (**b**) Classification of the detected signals into Maillard reaction products (square) and carbohydrate degradation products (circle). Glycine degradation products were not detected.

**Figure 2:**
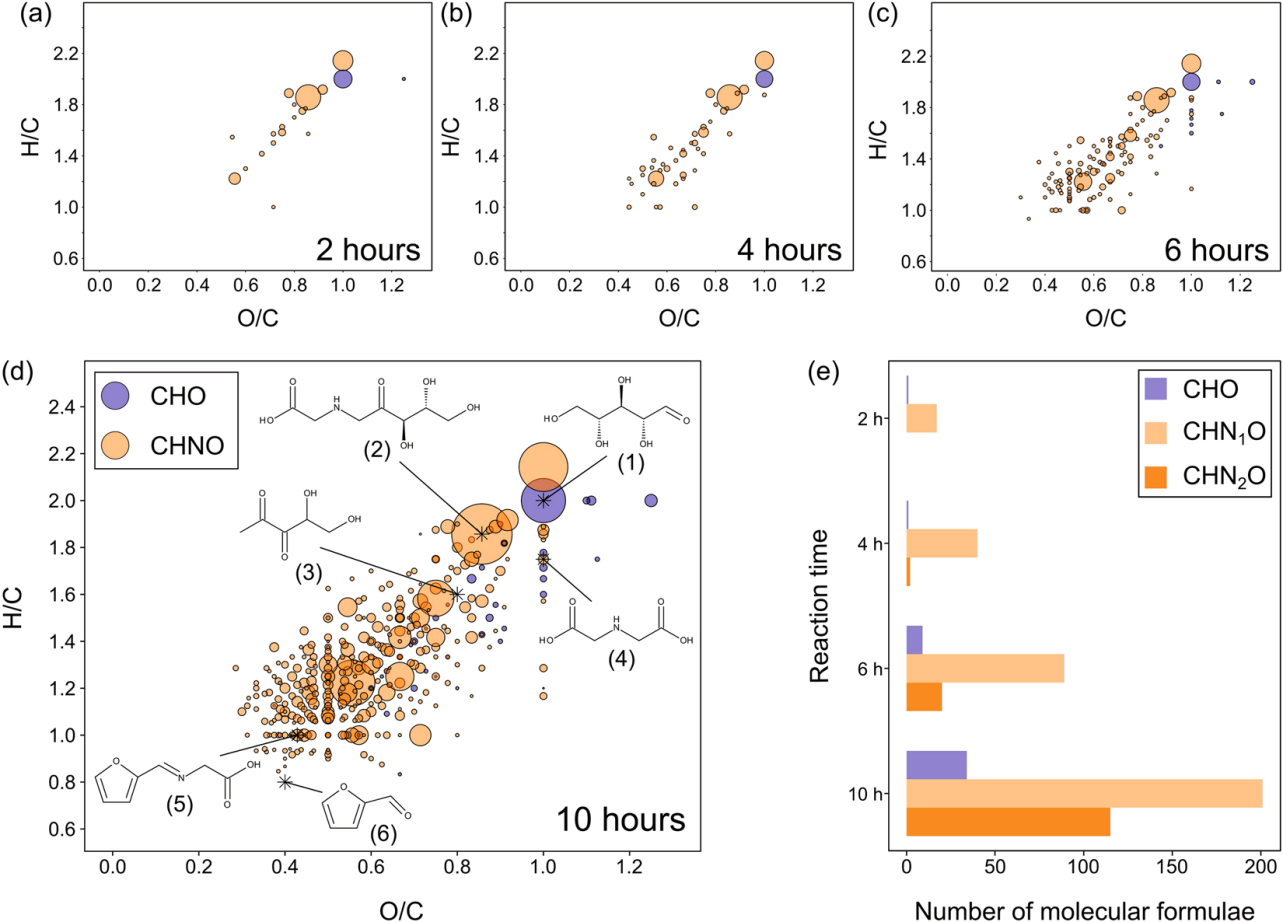
Compositional characteristics of MRPs and ribose. (**a–d**) Van Krevelen diagrams (H/C vs. O/C) were used to visualize the reaction progression. Features on imaginary lines with a slope of 2 indicate dehydration series, those on vertical and horizontal lines represent redox reaction series^16^. Selected known marker compounds illustrate the position in the diagrams depending on structural characteristics: ribose **1**, Amadori product (ARP, **2**), 1-deoxypentosone **3**, n-(carboxymethyl)glycine **4**, n-(2-furanylmethylene)glycine **5**, and furfural **6**. (**e**) The bar chart illustrates the absolute number of assigned molecular formulae for each reaction time classified into compositional spaces (CHO, CHN_1_O, and CHN_2_O).

Here, an equimolar mixture of ribose and glycine was heated under the same conditions L. C. Maillard used in his original experiments in 1912 (100 °C, unbuffered)^1^. We monitored the formation of MRPs over time in a non-targeted approach by FT-ICR-MS. Extracts of the model system were subjected to analysis using direct infusion FT-ICR-MS and a vast and complex pool of hundreds of distinct ion signals was observed (Fig. 1a). Starting with two initial reactants, the number of intermediates produced throughout the course of the MR increased with time. Reaction products were recorded using negative electrospray ionization to achieve higher selectivity for oxygen-rich analytes^12^ which are preferentially formed in the early MR. The instrument’s high resolving power (400000 at *m*/*z* 300) and mass accuracy enabled the assignment of the individual mass peaks to their corresponding unique elemental compositions. According to Yaylayan’s classification approach^13^ three kinds of reactions can occur when a sugar is heated in the presence of amino acids: (i) Degradation of the Amadori rearrangement product (ARP), Maillard reaction; (ii) carbohydrate degradation, caramelization type reactions; and (iii) amino acid degradation; (classification approach is described in the methods section). Formation of reactive intermediates which constantly feed the reaction pool, leads to an exponential increase in production of MRPs (Fig. 1b). After ten hours, we found hundreds of distinct molecular formulae which were not produced when ribose or glycine were heated alone. The shape of the curve (Fig. 1b) indicates that, even after ten hours, an end point of the reaction was not reached. By comparison, ribose caramelization (ribose heated alone; Fig. 1b) led to a linear increase in the number of decomposition products but only a few tens of compounds were formed. Sugar decomposition is known to predominantly occur at high temperatures (>120 °C) or under strongly alkaline or acidic conditions^14^ whereas the MR requires less energy^15^. Nevertheless, these ribose decomposition products (and some smaller ones outside our analytical window; <100 Da) may also be reactive intermediates which can contribute to the overall MR cascade. Most of the MRPs detected have a molecular mass below 400 Da. However, after two hours a large number of molecular formulae were found at a higher molecular weight than the initial ARP n-(1-deoxy-d-erythro-2-pentulos-1-yl)glycine (structure **2** in Fig. 2). Thus, MRPs are not only formed by degradation of the ARP into smaller molecules, also molecules of higher molecular weight are produced, which contribute to the diversity observed in the MR.

Molecular formulae retrieved for MRPs (and ribose) were projected into 2D van Krevelen plots^16^ (Fig. 2a–d). After two hours, the compound pool was almost exclusively made up of MRPs containing one nitrogen atom (Fig. 2e). After ten hours the number of detected CHNO and CHO compounds was 18 times as many as found after two hours (19 MRPs found after 2 h, 348 after 10 h). However, the formation of MRPs containing two nitrogen atoms showed a noticeably greater increase after six hours compared to MRPs containing only one or no nitrogen atoms. Starting with condensation of ribose (O/C: 1, H/C: 2) and glycine the MR begins in the top right corner of the van Krevelen diagrams. The initial condensation is followed by an extended series of dehydration reactions leading to MRPs with a higher degree of unsaturation and aromaticity (lower H/C and O/C ratios) as a function of time. After four hours, more than half of the MRPs produced were found in a very narrow range (1 ≤ H/C ≤ 1.5 and 0.35 ≤ O/C ≤ 0.65), so, although the number of MRPs produced is high, they are found in a very discrete chemical compositional space. Within the first four hours, we observed extended series of dehydration reactions were the most dominant in the formation of nitrogen-containing (CHNO) species. Interestingly, when nitrogen-free (CHO) compounds were considered separately, dehydration reactions did not play a significant role in the first six hours, neither in Maillard reactions (Fig. S4) nor when ribose was heated alone. This leads us to propose that dehydration of the carbohydrate backbone is favored when the sugar is covalently bound to amino compounds. These nitrogen-free (CHO) MRPs detected in the first six hours all revealed an average carbon oxidation state^17^ (OS_C_) higher than ribose (Fig. S4). Many of these intermediates may be formed by oxidative cleavage of dicarbonyl structures^18^. By comparison, the predominant CHNO-containing MRPs in the first four hours mostly revealed an OSc similar to that of the ARP (Fig. S5). Consequently, reactions of other type than oxidation reactions (*e.g*. dehydration) predominate the formation of CHNO-MRPs at the beginning of the MR. After six hours, both oxidized and reduced CHNO species were also observed. After ten hours, nitrogen-free (CHO) intermediates also showed pronounced dehydration series. By comparison, when ribose was heated alone, there was little evidence of dehydration reactions playing a meaningful role over the entire ten-hour cooking period. In this context, nitrogen-free (CHO) and nitrogen containing (CHNO) MRPs act very differently in their reactive behavior.

**Figure 3:**
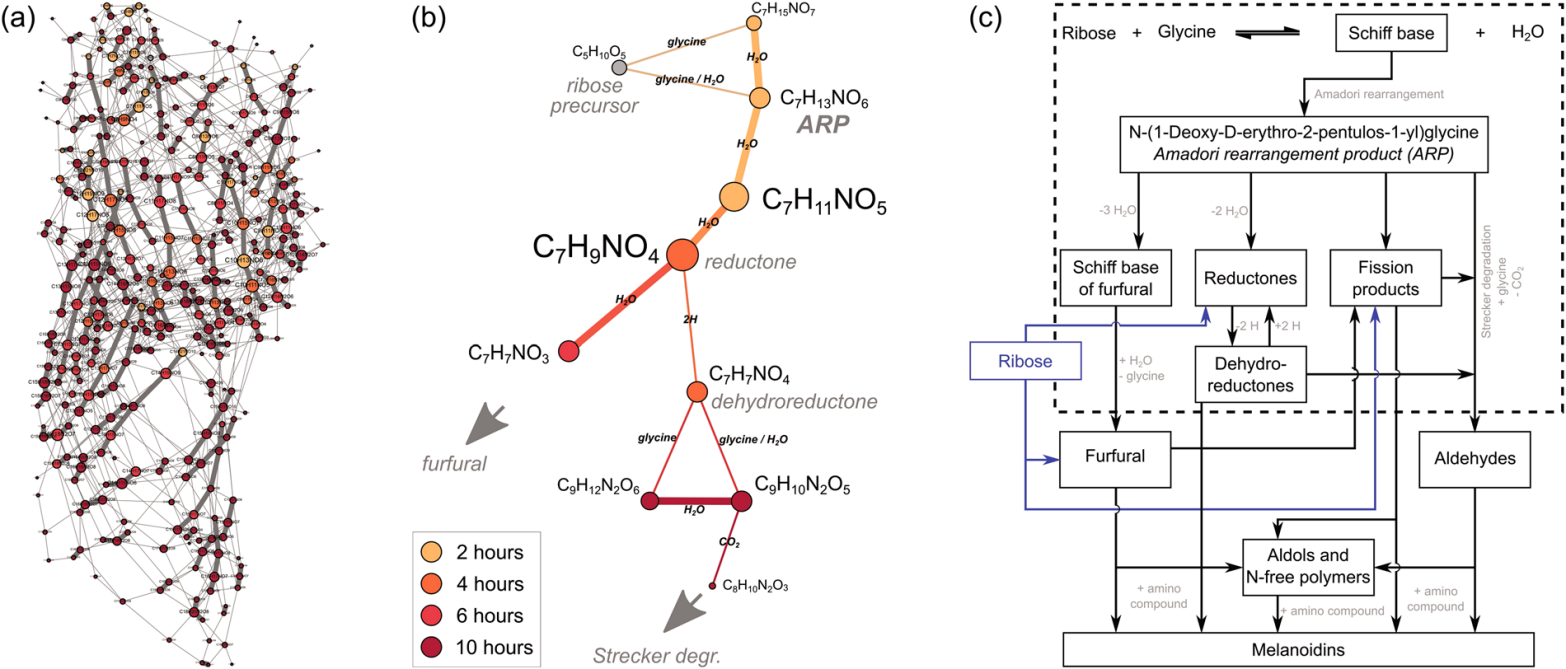
Time-resolved coherences between MRPs. (**a**) Mass difference network of MRPs, carbohydrate decomposition products and the ribose precursor. 98% of assigned ion signals (357/366) could be connected in the network by allowing only a set of seven simple transformations from the Hodge scheme: 2.01565 (2H), 12.00000 (Strecker degradation, +H_2_O/−CH_2_O), 18.01057 (H_2_O, in bold), 43.98983 (CO_2_), 57.02146 (glycine condensation), 75.03203 (glycine addition), 150.05283 (pentose addition). The transformations (edges) in the graph are undirected, reverse reactions are also possible. (**b**) The selection shows the coherences between observed molecular formulae as expected for the initial and intermediate phase by the reaction scheme (dashed box in **c**). (**c**) Fundamental Maillard reaction scheme adapted from Hodge^3^.

As all features which were detected after two, four, or six hours were still detectable in the samples heated for ten hours, the chemical pathways leading to these intermediates must be present in the mass spectra and can be characterized by studying the exact mass differences (MDs) between compounds which represent chemical transitions in the early stages of the MR such as dehydration, oxidation, *etc* ^3^. This type of data analysis shows the chemical relationships between the compounds and probes the data in a more reactivity-related context. It was possible to connect 98% of the observed compounds (MRPs, carbohydrate decomposition products, and ribose) with just seven types of transformations. A network graph was constructed (Fig. 3) where the nodes represent assigned molecular formulae which are connected to each other by accurate MDs representing possible chemical transformations. In fact, the network in Fig. 3a offers a broad picture of simple and repetitive reaction series occurring in parallel. Moreover, the decomposition of the ARP can be completely followed as a time dependent pathway until the formation of the furfural and Strecker imine (Fig. 3b). This confirms the comprehensive acquisition of early MRPs with the method applied. However, the ARP degradation as described in the Hodge scheme (Fig. 3c) constitutes only a small subpart of our network.

**Figure 4:**
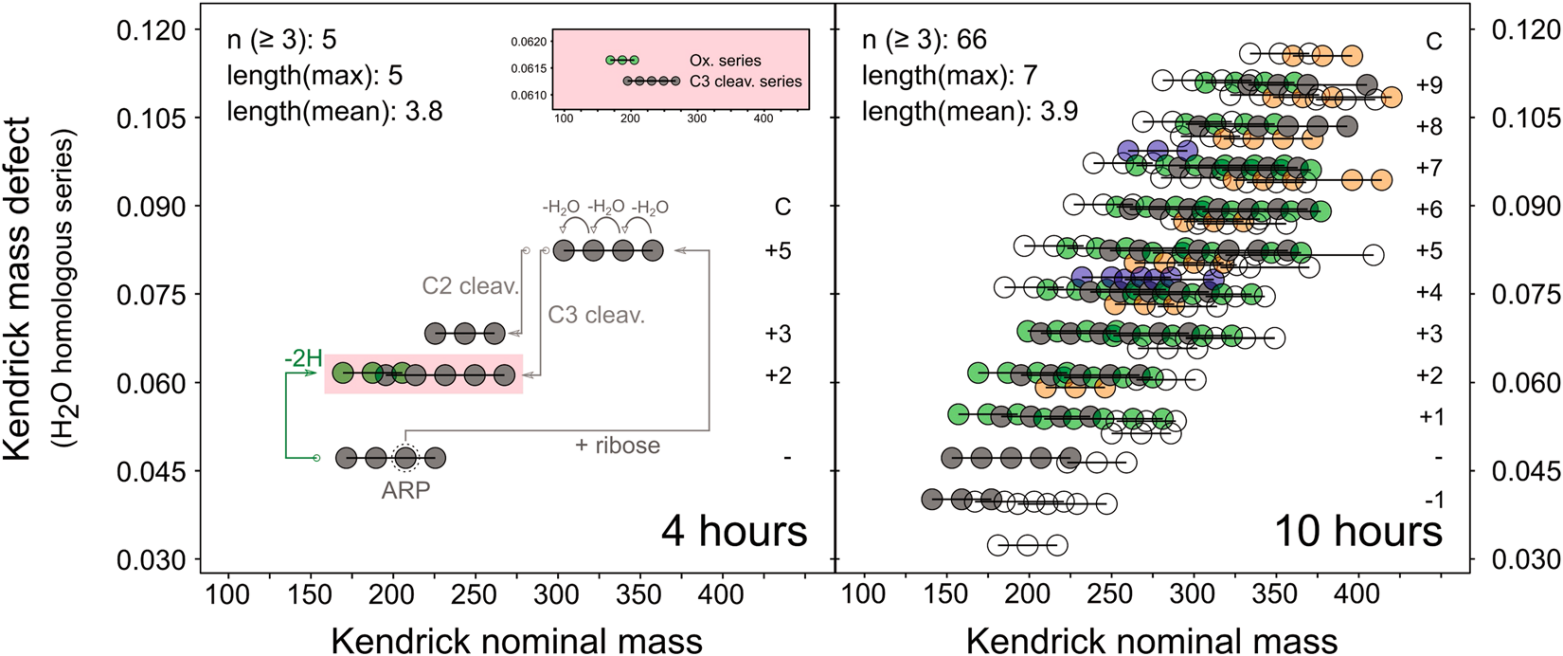
Visualization of dehydration series by Kendrick mass defect (KMD) analysis^16^. Conversion of the IUPAC mass to a Kendrick mass scale (IUPAC × 18/18.01057) projects dehydration series onto horizontal lines. Only series including at least three intermediates (n ≥ 3) are shown. Most series can be formed from the ARP by condensation with carbohydrate type (C_C_(H_2_O)_n_) fragments (grey), redox reactions (green) or a combination of both. Formation of nitrogen-free compounds (blue) and MRPs containing two nitrogen atoms (orange) must involve further release or addition/condensation of glycine.

In Fig. 4 we applied a modified Kendrick mass defect analysis^16^ in order to visualize the role of dehydration reactions as well as the relationships between each other. At the beginning, dehydration series are connected to each other by simple carbohydrate type fragments of type C_c_(H_2_O)_n_.

After the initial ribose-glycine condensation (2 h), the ARP was formed and subsequently dehydrated. At the same time, we observed another glycosylation of the ARP by C_5_H_10_O_5_ leading probably to n,n-bis(1-deoxy-d-erythro-2-pentulos-1-yl)-glycine. The formation of diketosamines has apparently been only reported for difructosamines. Mossine *et al*. showed that the degradation rate of difructosamines is much higher than for monofructosamines^19^. Consequently, the two further dehydration series (C +2 and C +3), detected after four hours, could arise from direct cleavage of dicarbonyl intermediates produced from the diketosamine. The amino intermediates formed from this reaction, potentially offer a new reducing end, which is able to undergo new glycine condensation, thus opening the CHN_2_O space, or aldol-type reactions with other carbonyl intermediates to extend the carbon backbone.

Little is known about C-C bond formation in the MR. The elongation of carbohydrates by aldol-type reactions is still under debate. However, intermolecular C-C bond formation between ketones and aldehydes may be catalyzed by the action of amino acids^20^. Recently, Pfeifer *et al*. reported an aldol-based polymerization of methylglyoxal produced during the decomposition of 3-deoxy-d-erythro-hexos-2-ulose leading to reactive aldehydes with extended carbon chains^21^. Additionally, oxidation of the ARP led to a further dehydration series (4 h). Both, fragmentation of the carbohydrate backbone and redox reactions, were found to be mainly responsible for the complete set of dehydration series found after ten hours. We found series ranging from ARP - C1 to ARP + C9. It is worth noting that all series greater than ARP + C5 must involve the formation of new C-C bonds in at least one stage of the reaction cascade and not only a breakdown into smaller fragments.

## Conclusion

In summary, this study emphasized the complexity of hundreds of distinct early MRPs produced in a simple two reactant system. Although isomers could not be resolved by our methods, we achieved a comprehensive coverage of intermediates involved in the early Maillard reaction. Those intermediates can be formed within a network of parallel reactions following repetitive patterns. Among others, dehydration, fragmentation of the carbohydrate backbone and redox reactions turned out to have a large impact on the diversity. Not only decomposition of the ARP but also the formation of initial intermediates with higher molecular weight than the ARP is involved in the reaction cascade. Extending these experiments by multiple amino or carbohydrate sources as in the case of food products would certainly amplify the diversity to new dimensions reaching thousands of distinct compounds.

## Methods

### Maillard model systems

D-(-)-Ribose (Rib, ≥99%) was purchased from Sigma-Aldrich (Steinheim, Germany). Glycine (Gly, ≥98.5%) was obtained from Merck (Darmstadt, Germany). Equimolar mixtures of ribose and glycine (0.1 M) were prepared in Milli-Q purified water (Millipore, Germany) immediately prior to thermal treatment. Samples were heated in a closed glass vial for two, four, six and ten hours (100 °C, waterbath). Additionally, blank samples containing only 0.1 M ribose or 0.1 M glycine were prepared. All experiments on Maillard model systems were carried out in triplicate (n = 3).

### Direct infusion FT-ICR mass spectrometry

Ultrahigh-resolution FT-ICR mass spectra were acquired with a 12 T Bruker Solarix mass spectrometer (Bruker Daltonics, Bremen, Germany) equipped with an APOLLO II electrospray source in negative ionization mode. For MS analysis the thermally processed Maillard model systems were diluted 1:500 (v/v) with methanol (LC-MS grade, Fluka, Germany). The diluted samples were infused into the electrospray ion source with a flow rate of 2 µL min^−1^. Settings for the ion source were: drying gas temperature 180 °C, drying gas flow 4.0 L min^−1^, capillary voltage 3600 V. Spectra were first externally calibrated by ion clusters of arginine (57 nmol mL^−1^ in methanol). Next, internal calibration of each spectrum was conducted with a reference list including selected Maillard reaction markers and ubiquitous fatty acids. The spectra were acquired with a time-domain of 4 megawords and 300 scans were accumulated within a mass range of *m*/*z* 92 to 1000. A resolving power of 400000 at *m*/*z* 300 was achieved. Raw spectra were post-processed by Compass DataAnalysis 4.2 (Bruker Daltonics, Bremen, Germany) and peaks with a signal-to-noise ratio (S/N) of at least 8 were exported to mass lists.

### Processing of FT-ICR-MS data

All exported *m*/*z* features were aligned in a matrix containing averaged *m/z* values (peak alignment window width: ±1 ppm, Fig. S1) and corresponding peak intensities of all analyzed samples^22^. Molecular formulae were assigned to the exact *m*/*z* values by mass difference network analysis using an in-house developed software tool^23^. For further processing, only those molecular formulae were considered which were found in all three replicates of at least one sample. In total, 373 detected features could be assigned to distinct and unique molecular formulae. More than 90% of all assignments were found within an error range of ±0.2 ppm (Fig. S2). All further calculations and filtering were done in Microsoft Excel 2010 and R Statistical Language (version 3.1.1)^24^.

### Classification into reaction pools

Molecular formulae were classified according to Yaylayan into three different reaction pools^13^: (i) Maillard reaction products (MRPs, Table S1), (ii) thermal induced carbohydrate degradation products, and (iii) amino acid degradation products. Ion signals which were found exclusively in all three replicates of the model systems but not in the blank samples (ribose and glycine heated alone) were classified as MRPs. Features also found in the ribose blank sample were classified as carbohydrate decomposition product. When signals were found in the model systems and both blank samples, they were considered to be contaminants. In total, eight different contaminants were detected known by us as trace contaminants omnipresent in solvents and sample preparation (Table S2). Glycine degradation products could not be detected by our analytical platform.

### Average carbon oxidation state

Carbon oxidation state of molecular formulae was calculated as suggested by Kroll *et al*.^17^:

$$\overline{O{S}_{C}}=-\sum _{i}OSi\,\frac{{n}_{i}}{{n}_{C}}$$

in which OS_c_ is determined by non-carbon atoms (H, O, and N) in the molecular formulae. The quotient n_i_/n_c_ is the molar ratio of element i to carbon and OS_i_ the oxidation state of element i set to +1, −2, and −3 for hydrogen, oxygen, and nitrogen, respectively.

## Electronic supplementary material

Supplementary Informationpdf
