# Supplementary material for: Evolution of Complex Maillard Chemical Reactions, Resolved in Time
Source: Sci Rep. 2017 Jun 12;7:3227. doi: 10.1038/s41598-017-03691-z (PMC5468300; doi:10.1038/s41598-017-03691-z)
Supplement: Supplementary file 1 — Supplementary Informationpdf [file 41598_2017_3691_MOESM1_ESM.pdf]

Supplementary Information

Evolution of Complex Maillard Chemical Reactions, Resolved in Time

Daniel Hemmler[1,2]*, Chloé Roullier-Gall[1,2], James W. Marshall[3], Michael Rychlik[1,2], Andrew J. Taylor[3], Philippe Schmitt-Kopplin[1,2]*

[1]Comprehensive Foodomics Platform, Analytical Food Chemistry, Technical University Munich, Alte Akademie 10, 85354 Freising, Germany. [2]Research Unit Analytical BioGeoChemistry (BGC), Helmholtz Zentrum München, Ingolstädter Landstrasse 1, 85764 Neuherberg, Germany. [3]The Waltham Centre for Pet Nutrition, Mars Petcare UK, Waltham-on-the-Wolds, Leicestershire., LE14 4RT, United Kingdom. *e-mail: schmitt-kopplin@helmholtz-muenchen.de; daniel.hemmler@tum.de

Table of contents

List of abbreviations 2
Preprocessing of FT-ICR-MS data 3
Classification into reaction pools 4
List of assigned molecular formulae 5
Average carbon oxidation state 10
References (Supplementary Information) 11

List of abbreviations

ARP	Amadori rearrangement product
FT-ICR-MS	Fourier transform ion cyclotron mass spectrometry
HMF	5-Hydroxymethylfurfural
KMD	Kendrick mass defect
MD	Mass difference
MR	Maillard reaction
MRP	Maillard reaction product
NMR	Nuclear magnetic resonance spectroscopy
OS_C	Average carbon oxidation state
TOF-MS	Time of flight mass spectrometry

Preprocessing of FT-ICR-MS data

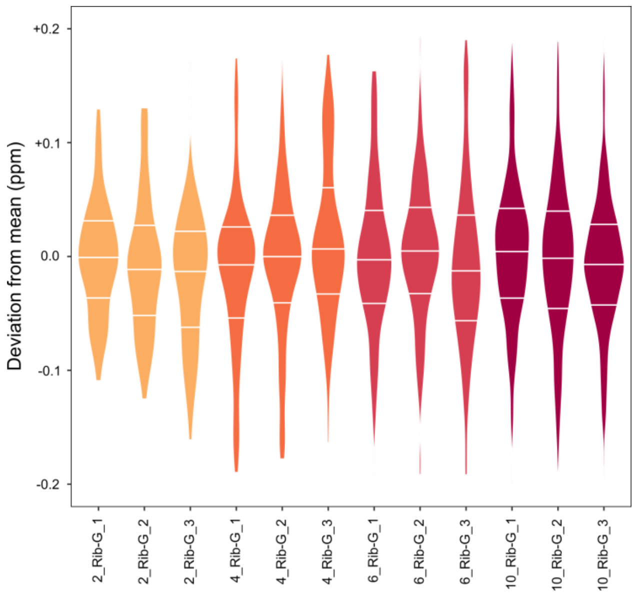

Figure S1. Peak alignment based on experimental *m/z* values with maximum of 1 ppm alignment window. Violin plots illustrate the quality of the alignment. Violins are horizontally divided into 25%, 50%, and 75% quantiles.

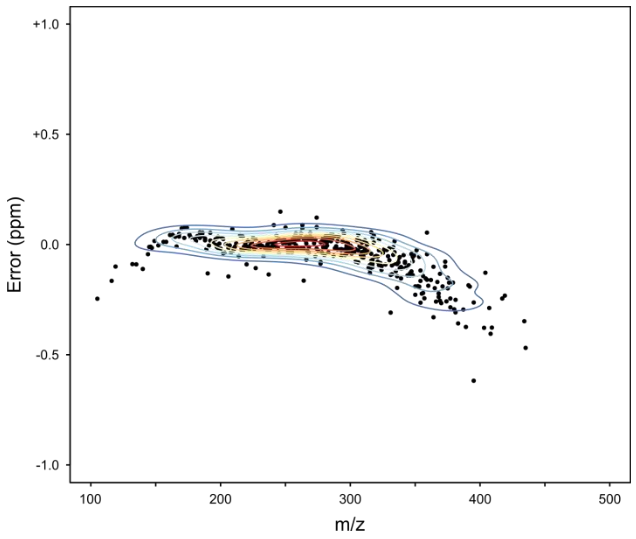

Figure S2. Error plot retrieved after molecular formula assignment. More than 90% of all molecular formulae were found within an error range of +/- 200 ppb, more than 75% within +/- 100 ppb.

Classification into reaction pools

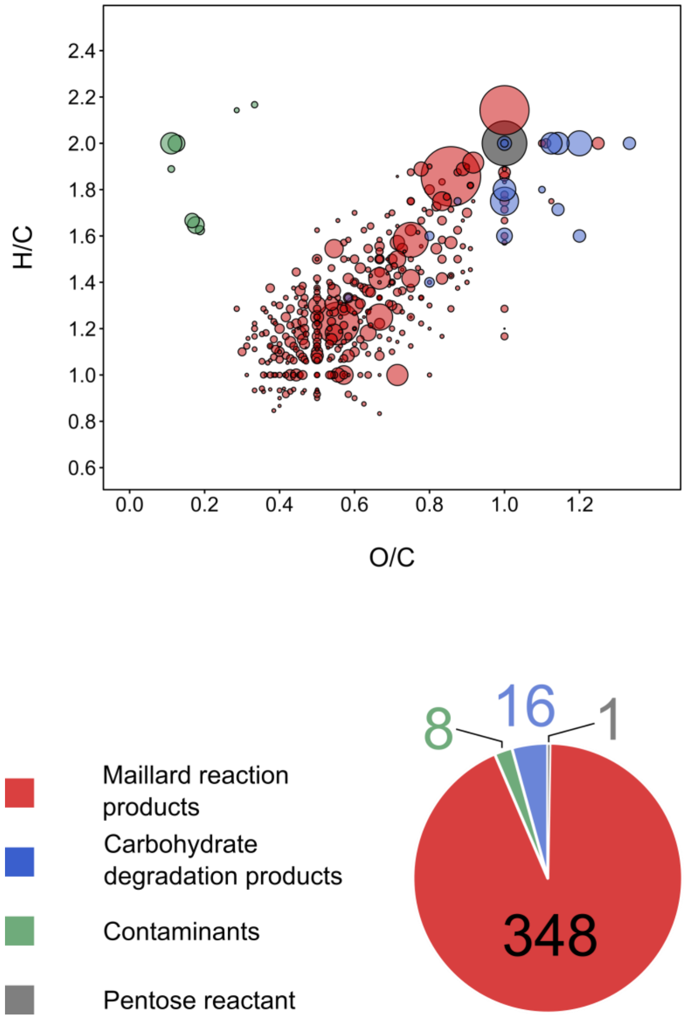

Figure S3. Classification of a ribose-glycine model system (10 h) into reaction pools according to the approach of Yaylayan[1].

List of assigned molecular formulae

Table S1. List of assigned molecular formulae classified as Maillard reaction products and ribose.

Peak no.	m/z	Error (ppm)	Molecular formula (neutral)	Time (h)	Peak no.	m/z	Error (ppm)	Molecular formula (neutral)	Time (h)
1	116.03530	-0.17	C4H7NO3	6	41	198.04081	0.04	C8H9NO5	6
2	132.03022	-0.09	C4H7NO4	6	42	198.07718	-0.02	C9H13NO4	6
3	135.02989	-0.09	C4H8O5	2	43	199.03605	0.00	C7H8N2O5	10
4	140.03530	-0.11	C6H7NO3	10	44	200.05644	-0.02	C8H11NO5	6
5	144.03023	-0.04	C5H7NO4	10	45	201.04047	0.03	C8H10O6	10
6	145.01425	-0.01	C5H6O5	10	46	202.03571	-0.04	C7H9NO6	6
7	146.04588	-0.01	C5H9NO4	2	47	202.07210	0.03	C8H13NO5	10
8	149.04555	0.02	C5H10O5	2	48	203.05611	-0.04	C8H12O6	10
9	152.03532	0.00	C7H7NO3	6	49	204.05136	0.00	C7H11NO6	2
10	156.03024	0.01	C6H7NO4	10	50	206.04585	-0.15	C10H9NO4	10
11	158.04588	0.01	C6H9NO4	6	51	206.06701	-0.02	C7H13NO6	2
12	161.04556	0.04	C6H10O5	10	52	207.05103	0.00	C7H12O7	6
13	166.05097	-0.01	C8H9NO3	10	53	208.06153	-0.04	C10H11NO4	6
14	168.03024	0.04	C7H7NO4	4	54	209.05680	0.07	C9H10N2O4	10
15	168.06663	0.05	C8H11NO3	6	55	210.04079	-0.02	C9H9NO5	4
16	170.04590	0.07	C7H9NO4	4	56	210.07718	-0.03	C10H13NO4	10
17	172.02515	0.03	C6H7NO5	10	57	211.07243	-0.01	C9H12N2O4	10
18	172.06155	0.08	C7H11NO4	10	58	212.05645	0.01	C9H11NO5	2
19	174.04081	0.08	C6H9NO5	6	59	214.03570	-0.05	C8H9NO6	6
20	176.05646	0.04	C6H11NO5	10	60	215.05612	0.04	C9H12O6	10
21	177.04047	0.03	C6H10O6	6	61	216.05136	-0.02	C8H11NO6	6
22	180.06662	0.03	C9H11NO3	10	62	216.08776	0.04	C9H15NO5	10
23	181.06187	0.02	C8H10N2O3	10	63	217.04662	0.04	C7H10N2O6	10
24	182.04589	0.04	C8H9NO4	6	64	218.03063	-0.01	C7H9NO7	10
25	184.02516	0.06	C7H7NO5	2	65	218.06701	-0.02	C8H13NO6	2
26	184.06154	0.03	C8H11NO4	6	66	219.05103	0.00	C8H12O7	6
27	185.04556	0.04	C8H10O5	10	67	220.04627	-0.02	C7H11NO7	10
28	186.04081	0.04	C7H9NO5	4	68	220.06151	-0.09	C11H11NO4	10
29	186.07719	0.05	C8H13NO4	10	69	220.08266	-0.01	C8H15NO6	6
30	188.02007	0.02	C6H7NO6	6	70	222.04079	-0.02	C10H9NO5	10
31	188.05645	0.03	C7H11NO5	2	71	222.06192	-0.02	C7H13NO7	6
32	189.04046	-0.02	C7H10O6	10	72	222.07718	-0.01	C11H13NO4	10
33	190.07207	-0.13	C7H13NO5	10	73	224.05645	0.01	C10H11NO5	4
34	191.05611	-0.01	C7H12O6	10	74	224.07758	-0.01	C7H15NO7	2
35	192.05137	0.02	C6H11NO6	10	75	225.05170	0.03	C9H10N2O5	10
36	192.06663	0.05	C10H11NO3	6	76	226.03571	-0.01	C9H9NO6	10
37	194.04589	0.01	C9H9NO4	4	77	226.07209	-0.02	C10H13NO5	4
38	196.02515	0.00	C8H7NO5	10	78	227.05609	-0.11	C10H12O6	10
39	196.06154	0.02	C9H11NO4	4	79	227.06735	0.02	C9H12N2O5	10
40	197.05678	-0.01	C8H10N2O4	6	80	228.05136	-0.01	C9H11NO6	4

Time: Reaction time when MRP was detected (SN >= 8) for the first time.

Table S1 (continued). List of assigned molecular formulae classified as Maillard reaction products and ribose.

Peak no.	m/z	Error (ppm)	Molecular formula (neutral)	Time (h)	Peak no.	m/z	Error (ppm)	Molecular formula (neutral)	Time (h)
81	228.08775	-0.01	C10H15NO5	6	121	256.08267	0.01	C11H15NO6	4
82	230.06701	0.01	C9H13NO6	4	122	257.06668	0.02	C11H14O7	10
83	231.05103	-0.01	C9H12O7	10	123	257.07792	0.04	C10H14N2O6	6
84	232.04628	-0.01	C8H11NO7	10	124	258.06194	0.05	C10H13NO7	6
85	233.06667	-0.05	C9H14O7	10	125	258.09831	0.00	C11H17NO6	2
86	234.06193	-0.01	C8H13NO7	6	126	259.04594	-0.02	C10H12O8	10
87	236.05644	-0.02	C11H11NO5	10	127	259.05717	-0.02	C9H12N2O7	10
88	236.07758	0.00	C8H15NO7	6	128	260.05646	0.03	C13H11NO5	10
89	237.05169	-0.02	C10H10N2O5	10	129	260.07758	0.02	C10H15NO7	4
90	237.08805	-0.14	C11H14N2O4	10	130	262.05686	0.05	C9H13NO8	10
91	238.03571	-0.01	C10H9NO6	10	131	262.07209	-0.02	C13H13NO5	10
92	238.07209	-0.02	C11H13NO5	4	132	262.09322	-0.02	C10H17NO7	10
93	240.05136	-0.01	C10H11NO6	6	133	263.06737	0.07	C12H12N2O5	10
94	240.08775	0.01	C11H15NO5	6	134	264.05137	0.03	C12H11NO6	10
95	241.04663	0.09	C9H10N2O6	10	135	264.07245	-0.16	C9H15NO8	10
96	241.08300	0.00	C10H14N2O5	10	136	264.08774	-0.02	C13H15NO5	10
97	242.06702	0.01	C10H13NO6	2	137	265.08300	0.02	C12H14N2O5	6
98	243.05103	-0.01	C10H12O7	10	138	266.06702	0.04	C12H13NO6	6
99	243.06227	0.04	C9H12N2O6	10	139	266.08814	0.01	C9H17NO8	4
100	244.04628	0.02	C9H11NO7	10	140	266.10338	-0.07	C13H17NO5	10
101	244.08266	-0.01	C10H15NO6	6	141	267.07215	-0.03	C9H16O9	6
102	245.06667	-0.04	C10H14O7	10	142	267.09865	0.02	C12H16N2O5	10
103	245.07792	0.02	C9H14N2O6	6	143	268.04627	-0.04	C11H11NO7	10
104	246.06193	0.02	C9H13NO7	10	144	268.08266	-0.01	C12H15NO6	4
105	246.09835	0.15	C10H17NO6	10	145	269.07793	0.05	C11H14N2O6	6
106	248.05645	0.01	C12H11NO5	10	146	270.06193	0.02	C11H13NO7	4
107	248.07758	0.01	C9H15NO7	4	147	270.09832	0.03	C12H17NO6	10
108	249.06160	0.02	C9H14O8	10	148	272.07758	0.01	C11H15NO7	4
109	249.08809	0.02	C12H14N2O4	10	149	273.06158	-0.05	C11H14O8	10
110	250.07212	0.08	C12H13NO5	10	150	273.07283	0.02	C10H14N2O7	10
111	250.09323	0.01	C9H17NO7	2	151	274.05688	0.12	C10H13NO8	10
112	251.06734	-0.01	C11H12N2O5	10	152	274.07209	-0.02	C14H13NO5	10
113	252.05135	-0.04	C11H11NO6	6	153	274.09325	0.08	C11H17NO7	6
114	252.07249	-0.03	C8H15NO8	4	154	275.07724	-0.03	C11H16O8	10
115	252.08775	0.01	C12H15NO5	6	155	277.05649	-0.09	C10H14O9	10
116	253.05651	0.00	C8H14O9	6	156	277.08297	-0.08	C13H14N2O5	10
117	253.08299	-0.02	C11H14N2O5	10	157	277.11939	0.01	C14H18N2O4	10
118	254.06702	0.01	C11H13NO6	4	158	278.06700	-0.04	C13H13NO6	6
119	255.05102	-0.04	C11H12O7	10	159	278.08815	0.01	C10H17NO8	2
120	256.04627	-0.02	C10H11NO7	10	160	279.06227	0.03	C12H12N2O6	10

Time: Reaction time when MRP was detected (SN >= 8) for the first time.

Table S1 (continued). List of assigned molecular formulae classified as Maillard reaction products and ribose.

Peak no.	m/z	Error (ppm)	Molecular formula (neutral)	Time (h)	Peak no.	m/z	Error (ppm)	Molecular formula (neutral)	Time (h)
161	279.09864	-0.01	C13H16N2O5	10	201	300.10888	-0.01	C13H19NO7	10
162	280.04628	0.01	C12H11NO7	10	202	301.08299	-0.04	C15H14N2O5	6
163	280.08267	0.02	C13H15NO6	6	203	301.10412	-0.01	C12H18N2O7	10
164	280.10378	-0.05	C10H19NO8	10	204	302.06701	-0.02	C15H13NO6	10
165	281.03029	-0.02	C12H10O8	10	205	302.08814	-0.03	C12H17NO8	2
166	281.07792	0.04	C12H14N2O6	6	206	303.08338	-0.03	C11H16N2O8	4
167	282.06193	0.01	C12H13NO7	6	207	304.08266	-0.02	C15H15NO6	10
168	282.08307	0.04	C9H17NO9	10	208	304.10377	-0.08	C12H19NO8	10
169	282.09831	-0.01	C13H17NO6	10	209	305.11430	0.02	C15H18N2O5	10
170	283.09355	-0.04	C12H16N2O6	10	210	306.06190	-0.08	C14H13NO7	10
171	285.03645	0.04	C10H10N2O8	10	211	306.08305	-0.03	C11H17NO9	6
172	285.07284	0.05	C11H14N2O7	10	212	306.09833	0.04	C15H17NO6	10
173	285.08273	0.02	C9H18O10	6	213	307.06708	0.02	C11H16O10	10
174	285.10922	0.02	C12H18N2O6	10	214	307.09356	-0.01	C14H16N2O6	6
175	286.05685	0.02	C11H13NO8	10	215	308.07758	-0.01	C14H15NO7	6
176	286.09324	0.05	C12H17NO7	6	216	308.09869	-0.05	C11H19NO9	10
177	287.08846	-0.05	C11H16N2O7	10	217	309.06158	-0.06	C14H14O8	10
178	288.07250	0.04	C11H15NO8	6	218	309.07282	-0.03	C13H14N2O7	10
179	288.10887	-0.02	C12H19NO7	10	219	309.10922	0.01	C14H18N2O6	6
180	289.08299	-0.01	C14H14N2O5	10	220	310.05683	-0.05	C13H13NO8	10
181	290.06701	-0.02	C14H13NO6	10	221	310.09321	-0.06	C14H17NO7	6
182	290.08815	0.02	C11H17NO8	6	222	311.08848	0.02	C13H16N2O7	10
183	291.06227	0.04	C13H12N2O6	10	223	311.09837	-0.01	C11H20O10	10
184	292.08266	-0.02	C14H15NO6	10	224	312.07250	0.01	C13H15NO8	10
185	293.07790	-0.03	C13H14N2O6	10	225	312.10887	-0.04	C14H19NO7	10
186	293.11428	-0.05	C14H18N2O5	10	226	313.10412	-0.03	C13H18N2O7	10
187	294.06193	0.01	C13H13NO7	10	227	314.08811	-0.11	C13H17NO8	6
188	294.08306	0.02	C10H17NO9	6	228	315.08336	-0.11	C12H16N2O8	10
189	294.09833	0.07	C14H17NO6	10	229	315.09326	-0.09	C10H20O11	10
190	295.05717	-0.02	C12H12N2O7	10	230	315.09861	-0.13	C16H16N2O5	10
191	295.06707	-0.02	C10H16O10	6	231	316.06743	0.06	C12H15NO9	10
192	295.09358	0.05	C13H16N2O6	6	232	316.08264	-0.07	C16H15NO6	10
193	296.07756	-0.05	C13H15NO7	6	233	316.10378	-0.04	C13H19NO8	4
194	296.09869	-0.05	C10H19NO9	10	234	317.07789	-0.07	C15H14N2O6	10
195	297.07282	-0.02	C12H14N2O7	10	235	317.08778	-0.10	C13H18O9	10
196	297.10920	-0.04	C13H18N2O6	10	236	317.09901	-0.09	C12H18N2O8	6
197	298.05683	-0.03	C12H13NO8	6	237	317.11431	0.03	C16H18N2O5	10
198	298.09323	0.01	C13H17NO7	4	238	318.08306	0.01	C12H17NO9	4
199	299.08847	-0.02	C12H16N2O7	4	239	318.09833	0.04	C16H17NO6	10
200	300.07249	-0.02	C12H15NO8	4	240	319.09355	-0.05	C15H16N2O6	10

Time: Reaction time when MRP was detected (SN >= 8) for the first time.

Table S1 (continued). List of assigned molecular formulae classified as Maillard reaction products and ribose.

Peak no.	m/z	Error (ppm)	Molecular formula (neutral)	Time (h)	Peak no.	m/z	Error (ppm)	Molecular formula (neutral)	Time (h)
241	320.09869	-0.05	C12H19NO9	2	281	343.09353	-0.10	C17H16N2O6	10
242	321.07281	-0.05	C14H14N2O7	10	282	344.09866	-0.14	C14H19NO9	6
243	321.10919	-0.08	C15H18N2O6	6	283	345.10918	-0.09	C17H18N2O6	10
244	322.07794	-0.09	C11H17NO10	10	284	346.09318	-0.13	C17H17NO7	10
245	322.09322	-0.03	C15H17NO7	6	285	346.11431	-0.14	C14H21NO9	10
246	322.11437	0.02	C12H21NO9	10	286	347.08846	-0.04	C16H16N2O7	10
247	323.08845	-0.08	C14H16N2O7	6	287	347.12482	-0.12	C17H20N2O6	10
248	324.07246	-0.12	C14H15NO8	10	288	348.07247	-0.08	C16H15NO8	10
249	324.10885	-0.10	C15H19NO7	10	289	348.09360	-0.06	C13H19NO10	10
250	325.10411	-0.04	C14H18N2O7	6	290	348.12996	-0.14	C14H23NO9	10
251	326.08814	-0.02	C14H17NO8	10	291	349.10411	-0.05	C16H18N2O7	10
252	327.08336	-0.09	C13H16N2O8	10	292	350.08806	-0.23	C16H17NO8	10
253	328.06740	-0.03	C13H15NO9	10	293	350.10921	-0.19	C13H21NO10	10
254	329.09902	-0.05	C13H18N2O8	10	294	351.08338	-0.03	C15H16N2O8	10
255	330.08305	-0.02	C13H17NO9	10	295	352.10373	-0.19	C16H19NO8	10
256	330.11942	-0.07	C14H21NO8	10	296	353.09902	-0.06	C15H18N2O8	10
257	331.07821	-0.31	C12H16N2O9	10	297	353.13536	-0.19	C16H22N2O7	10
258	331.09352	-0.14	C16H16N2O6	10	298	354.08300	-0.17	C15H17NO9	10
259	332.07753	-0.14	C16H15NO7	10	299	354.10415	-0.12	C12H21NO11	10
260	332.09868	-0.10	C13H19NO9	10	300	354.11935	-0.26	C16H21NO8	10
261	333.07282	-0.01	C15H14N2O7	10	301	355.11461	-0.22	C15H20N2O8	10
262	333.10920	-0.05	C16H18N2O6	10	302	356.09863	-0.22	C15H19NO9	10
263	334.07794	-0.09	C12H17NO10	6	303	356.11979	-0.14	C12H23NO11	2
264	334.09322	-0.04	C16H17NO7	10	304	357.09387	-0.24	C14H18N2O9	10
265	334.11436	0.02	C13H21NO9	10	305	358.11430	-0.15	C15H21NO9	10
266	335.08844	-0.12	C15H16N2O7	10	306	359.08850	0.05	C17H16N2O7	10
267	336.07246	-0.09	C15H15NO8	10	307	359.10955	-0.16	C14H20N2O9	10
268	336.09356	-0.20	C12H19NO10	6	308	359.12485	-0.04	C18H20N2O6	10
269	336.10883	-0.14	C16H19NO7	10	309	360.09356	-0.19	C14H19NO10	10
270	337.06772	-0.08	C14H14N2O8	6	310	362.10922	-0.16	C14H21NO10	2
271	337.10411	-0.06	C15H18N2O7	6	311	364.10376	-0.10	C17H19NO8	10
272	338.08812	-0.06	C15H17NO8	10	312	364.12480	-0.33	C14H23NO10	10
273	338.10924	-0.11	C12H21NO10	2	313	365.09897	-0.20	C16H18N2O8	10
274	339.08336	-0.10	C14H16N2O8	10	314	366.08296	-0.26	C16H17NO9	10
275	339.10447	-0.15	C11H20N2O10	10	315	367.11463	-0.17	C16H20N2O8	10
276	339.11974	-0.11	C15H20N2O7	10	316	368.09863	-0.22	C16H19NO9	10
277	340.10376	-0.11	C15H19NO8	10	317	368.11975	-0.24	C13H23NO11	2
278	341.09903	-0.05	C14H18N2O8	10	318	369.09386	-0.26	C15H18N2O9	10
279	342.08303	-0.08	C14H17NO9	10	319	369.13029	-0.13	C16H22N2O8	10
280	342.11942	-0.08	C15H21NO8	10	320	370.11426	-0.27	C16H21NO9	10

Time: Reaction time when MRP was detected (SN >= 8) for the first time.

Table S1 (continued). List of assigned molecular formulae classified as Maillard reaction products and ribose.

Peak no.	m/z	Error (ppm)	Molecular formula (neutral)	Time (h)	Peak no.	m/z	Error (ppm)	Molecular formula (neutral)	Time' (h)
321	371.10954	-0.19	C15H20N2O9	10	336	389.09890	-0.37	C18H18N2O8	10
322	373.10409	-0.10	C18H18N2O7	10	337	391.11462	-0.19	C18H20N2O8	10
323	373.14048	-0.08	C19H22N2O6	10	338	392.11976	-0.19	C15H23NO11	10
324	374.10918	-0.26	C15H21NO10	10	339	395.10936	-0.62	C17H20N2O9	10
325	375.10447	-0.15	C14H20N2O10	6	340	395.13063	-0.26	C14H24N2O11	10
326	375.11970	-0.20	C18H20N2O7	10	341	403.11454	-0.38	C19H20N2O8	10
327	376.12486	-0.17	C15H23NO10	6	342	404.11979	-0.13	C16H23NO11	10
328	377.09895	-0.25	C17H18N2O8	10	343	407.10949	-0.29	C18H20N2O9	10
329	377.13532	-0.29	C18H22N2O7	10	344	408.15097	-0.41	C16H27NO11	10
330	379.11463	-0.17	C17H20N2O8	10	345	409.12510	-0.38	C18H22N2O9	10
331	380.09860	-0.29	C17H19NO9	10	346	417.13024	-0.25	C20H22N2O8	10
332	381.09384	-0.31	C16H18N2O9	10	347	419.13064	-0.23	C16H24N2O11	10
333	381.13025	-0.25	C17H22N2O8	10	348	434.13025	-0.35	C17H25NO12	10
334	383.10947	-0.36	C16H20N2O9	10	349	435.14070	-0.47	C20H24N2O9	10
335	387.11966	-0.30	C19H20N2O7	10					

Time: Reaction time when MRP was detected (SN >= 8) for the first time.

Table S2. List of detected contaminants.

m/z	Error (ppm)	Molecular formula (neutral)	Source
255.23296	0.02	C16H32O2	Fatty acid 16:0
265.14791	0.02	C12H26O4S	Alkyl sulfate
281.24861	0.03	C18H34O2	Fatty acid 18:1
283.26426	0.02	C18H36O2	Fatty acid 18:0
293.1792	-0.02	C14H30O4S	Alkyl sulfate
297.15299	0.01	C16H26O3S	Benzenesulfonic acid
311.16863	-0.02	C17H28O3S	Benzenesulfonic acid
325.18428	-0.03	C18H30O3S	Benzenesulfonic acid

Average carbon oxidation state

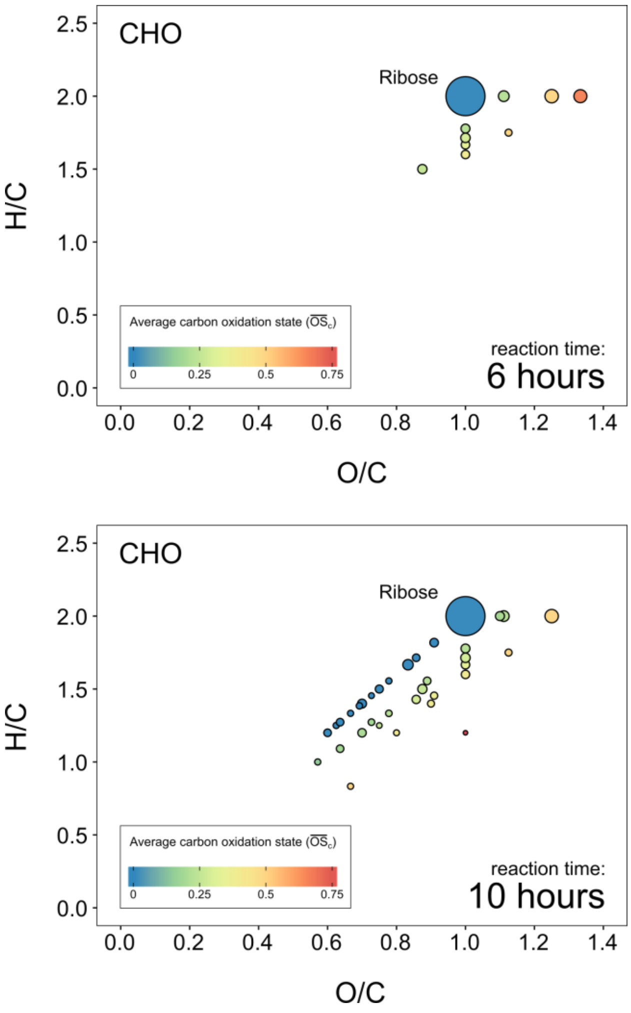

Figure S4. Van Krevelen diagrams (H/C vs. O/C) for nitrogen-free MRPs detected after six (top) and ten hours (bottom). Color code illustrates the average carbon oxidation state (OS_C). Bubble size is scaled to relative peak intensity. $C_3H_6O_4$ (probably glyceric acid) was only detected in the Maillard model systems after six hours. However, it could also be produced when ribose was heated alone for ten hours.

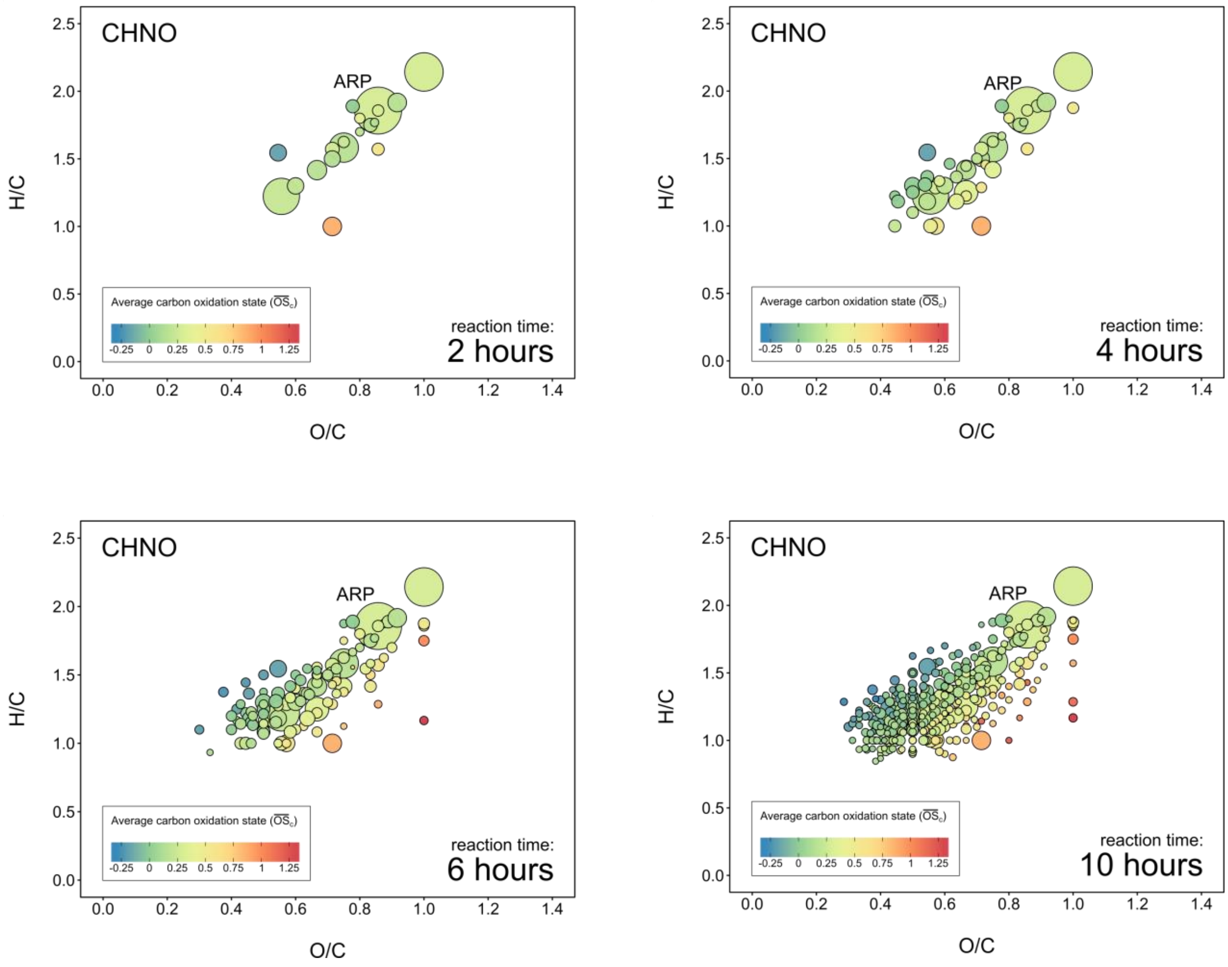

Figure S5. Van Krevelen diagrams (H/C vs. O/C) for nitrogen-containing MRPs detected at four different reaction times (two, four, six, and ten hours). Color code illustrates the average carbon oxidation state (OS_C). Bubble size is scaled to relative peak intensity.

References (Supplementary Information)

1. Yaylayan, V.A. Classification of the Maillard reaction: A conceptual approach. *Trends Food Sci. Technol.* **8,** 13–18 (1997).
